# Intra-abdominal Pressures during Voluntary and Reflex Cough

**DOI:** 10.1186/1745-9974-4-2

**Published:** 2008-04-30

**Authors:** W Robert Addington, Robert E Stephens, Michael M Phelipa, John G Widdicombe, Robin R Ockey

**Affiliations:** 1 W. Robert Addington, D.O., 101 E. Florida Avenue, Melbourne, FL, 32901, 321-984-4628, USA; 2 Department of Anatomy, Kansas City University of Medicine and Biosciences, Kansas City, MO, USA; 3 Melbourne, FL, USA; 4 116 Pepys Road, London SW208NY, UK; 5 Orem, UT, USA

## Abstract

**Background:**

Involuntary coughing such as that evoked from the larynx, the laryngeal cough reflex (LCR), triggers a coordinated contraction of the thoracic, abdominal and pelvic muscles, which increases intra-abdominal pressure (IAP), displaces the diaphragm upwards and generates the expiratory force for cough and airway clearance. Changes in the IAP during voluntary cough (VC) and the LCR can be measured via a pressure catheter in the bladder. This study evaluated the physiological characteristics of IAP generated during VC and the LCR including peak and mean pressures and calculations of the area under the curve (AUC) values during the time of the cough event or epoch.

**Methods:**

Eleven female subjects between the ages of 18 and 75 underwent standard urodynamic assessment with placement of an intravesicular catheter with a fiberoptic strain gauge pressure transducer. The bladder was filled with 200 ml of sterile water and IAP recordings were obtained with VC and the induced reflex cough test (RCT) using nebulized inhaled 20% tartaric acid to induce the LCR. IAP values were used to calculate the area under the curve (AUC) by the numerical integration of intravesicular pressure over time (cm H_2_O·s).

**Results:**

The mean (± SEM) AUC values for VC and the LCR were 349.6 ± 55.2 and 986.6 ± 116.8 cm H_2_O·s (p < 0.01). The mean IAP values were 45.6 ± 4.65 and 44.5 ± 9.31 cm H_2_O (NS = .052), and the peak IAP values were 139.5 ± 14.2 and 164.9 ± 15.8 cm H_2_O (p = 0.07) for VC and LCR, respectively.

**Conclusion:**

The induced LCR is the involuntary rapid and repeated synchronous expiratory muscle activation that causes and sustains an elevated IAP over time, sufficient for airway protection. VC and LCR have different neurophysiological functions. Quantification of the LCR using AUC values and mean or peak IAP values may be useful as a clinical tool for determining neurophysiological airway protection status and provide a quantitative assessment of changes in a patient's functional recovery or decline.

## Introduction

Neurophysiological protection of the upper airway is a critical function of the laryngeal cough reflex (LCR). Coughing involves coordinated contractions of the thoracic, abdominal and pelvic muscles. On videofluoroscopy, reflex cough (RC) caused increased upward displacement of the diaphragm as compared with voluntary cough (VC) [1]. This diaphragmatic displacement is a result of the contraction of the external abdominal obliques, intercostals and associated expiratory muscles. The force of these contractions compresses the abdominal viscera and proportionately displaces the diaphragm superiorly, almost to mid-sternal levels in reflex cough, but not for VC. These contractions cause an increase in intra-abdominal pressure (IAP), which is synchronized with urethral and rectal closure to prevent incontinence.

Although different patterns of "cough" have been described; the "classical" definition of cough starts with an inspiration, which is followed by compressive and expulsive phases; and is either a brainstem reflex or a cortically mediated response characteristic of VC. VC appears to play a role in clearing the vocal cords during speech [2]. However, the expiration reflex is a brainstem mediated reflex that initiates an immediate series of expiratory efforts without an inspiratory phase precedes the noxious stimulus. This type of cough is characterized by a synchronous series of expiratory reflex coughs with a short latency [3-5], and has a role in clearing the upper airway of potential aspirants during inhalation and swallowing [6]. Increased IAP provides the expiratory force for the protective airway clearing function of the LCR and producing a VC). This distinction is physiologically important because the two types of reflex differ in neurophysiological and pharmacological mechanisms [6-8].

Previously, it has not been possible to reliably analyze the quantitative changes in the IAP associated with VC and the LCR. The changes in IAP during cough may be measured using pressure catheters in the bladder and/or rectum. Since quantitative measurement of changes in IAP during VC and reflex cough may be useful in the clinical setting, this investigation was designed to assess VC and LCR IAPs using intravesicular pressure catheters and urodynamic analysis of pressure changes.

This study evaluated changes in the IAP during VC and the LCR as indicated by the measurements of the mean and peak IAPs, and mathematical calculations of the area under the curve (AUC, pressure·time) values during VC and LCR cough epochs.

## Materials and methods

Following informed consent, eleven female subjects between the ages of 18 and 75 were enrolled. Nine subjects had complaints of mild stress urinary incontinence without any neurological history. One subject (subject 10) had multiple sclerosis (MS) and was non-ambulatory with internuclear ophthalmoplegia and neurological deficits associated with cranial nerves II, III, IV and VI, but no history of pneumonia. A further subject (subject 11) was tested 8 weeks after sustaining a T_4 _complete spinal cord injury (SCI) and therefore had serious loss of control of her expiratory muscles; her results are mentioned briefly but are not included in the statistical analyses.

Evaluations were performed with a multi-channel urodynamic (UD) system that used a fiber-optic, disposable strain gauge pressure transurethral bladder catheter and a rectal catheter. With sterile technique, the calibrated bladder catheter was placed and secured to the subject's thigh. With continuous dual-channel recording, the subject's bladder was filled slowly with sterile water until 200 ml had been introduced.

**Figure 1:**
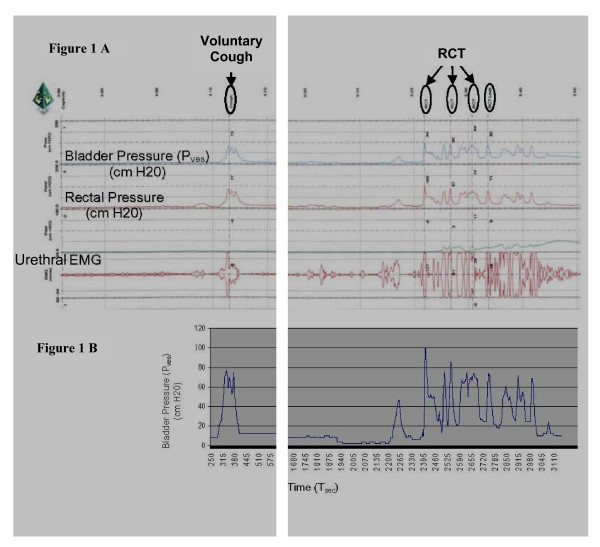
**A**. An urodynamic (UD) tracing (on a compressed timeline) of a subject demonstrating voluntary cough and an episode of RCT coughs (i.e., LCR) triggered by the RCT. A pressure sensor catheter was inserted into the subject's bladder and rectum, and the bladder was filled to 200 ml using sterile saline. Intravesicular bladder pressure was recorded at 30 samples per second. Subject was asked to voluntarily cough and the RCT was performed. Each cough episode was traced and the coordinates corresponding to a particular bladder pressure measurement (P_ves_) and the IAP at that time (T_sec_) were recorded for each peak, valley and slope change of the pressure tracing. **B**. A record was made of the complete cough episode timeline. As a part of this process, maximal IAP for each cough event was determined. Interpolation was used to fill in the remaining P_ves _between each annotated point. The average P_ves _was then calculated for each second of the timeline, and plotted as a pressure versus time graph of the cough episode.

Subjects were asked to deeply inhale and perform strong voluntary coughs, which were recorded on the UD system. Tartaric acid-induced reflex cough test (RCT) was used to elicit a LCR in all subjects [2,5,9-18]. The RCT used a jet nebulized concentration of 20% L-(+)-tartaric acid dissolved in 0.15 mM sterile NaCl solution (Nephron Pharmaceuticals, Orlando, FL). The jet nebulizer was activated with 50 psi from a tank that produced an average droplet diameter of 1–2 microns or less. During the RCT, the subject was asked to exhale completely, the nostrils were pinched closed, the nebulizer mouthpiece was placed in the mouth and subjects sealed the mouthpiece with their lips during the brisk inhalation. The RCT normally causes an immediate episode of several coughs. During VC and LCR, the intravesicular (bladder) pressure, rectal pressure and urethral EMG were also recorded for all subjects. (Fig. 1A) [19].

### Analysis of the IAP

Graphs from the original urodynamic assessment were digitized and the IAPs generated during the cough were quantified (Fig. 1B). Each cough epoch was analyzed throughout its duration. Deviation from baseline intra-abdominal pressure defined the start of the cough episode. The end of the cough epoch could be noted on the UD tracing as the IAP returned to nearly baseline levels. An analysis of the IAP rate of change indicated that an effective sampling rate of 30 samples/sec was appropriate for further analysis. The IAP was measured at this rate for each subject from the continuous UD recording. A graphic recording of pressure with vertical time lines was used to determine the peak IAPs (maximum intravesicular pressure during each expiratory cough effort), the mean IAP (over the period of the expiratory cough efforts), the durations of the cough epochs, the number of IAP peaks and the peak values for each cough epoch, and to derive the AUC values during each cough epoch. In this study, AUC is a product of pressure and time, expressed as cm H_2_O·s.

**Figure 2:**
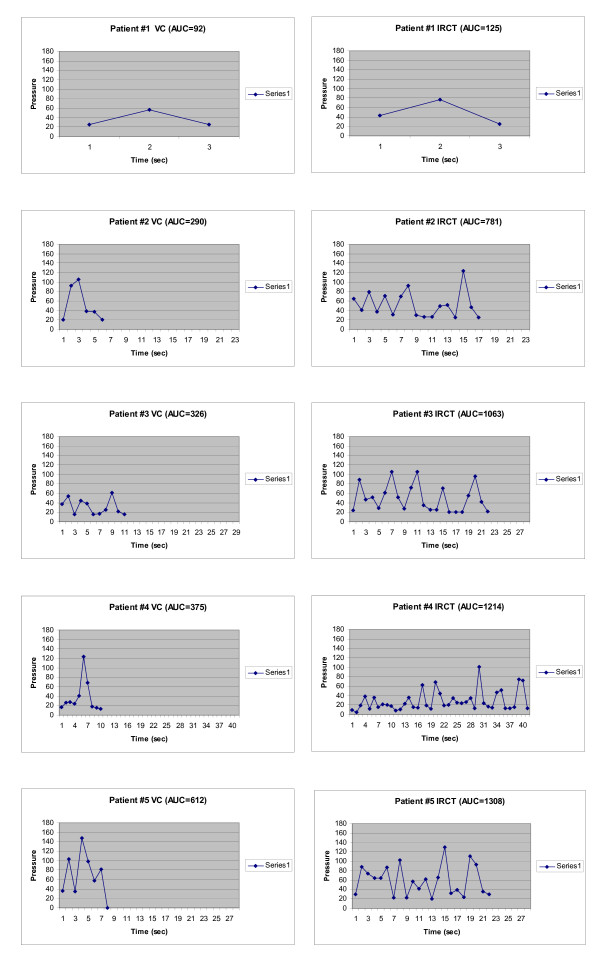
Area under the Curve Graphs for Subjects 1–5.

**Figure 3:**
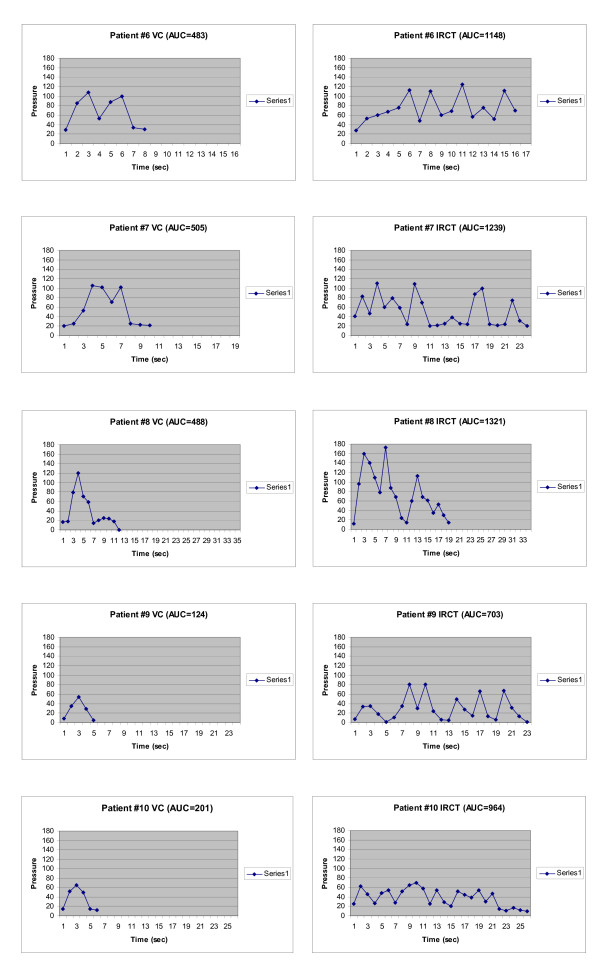
Area under the Curve Graphs for Subjects 6–10. Subject 10 had SUI and multiple sclerosis.

The UD tracing for each cough epoch was quantified and the coordinates corresponding to a particular IAP measurement and the IAP at that time were recorded for each peak, valley and slope change of the pressure tracing. A record was made of the complete cough epoch timeline (Fig. 2 and Fig. 3). Each second of the timeline was divided into 30 equal parts, i.e., 30 samples/s. The remaining pressures were interpolated between each annotated point. The mean IAP was then calculated for each second of the timeline, and plotted as a pressure versus time graph of the cough epoch (Fig. 2 and Fig. 3).

From the mean IAP values, AUC values were then calculated by the numerical integration of intravesicular pressure over time using Boole's rule [20]. Due to the diminished cough response and data points available for analysis, Simpson's 3/8 rule was the appropriate formula for the subject 11, who had a T_4 _complete spinal cord injury (SCI) and an abnormal LCR [20]. A paired t-test was used to compare the AUC values, mean IAP and peak IAP values for VC and LCR responses using SPSS statistical software (version 10.0.5).

## Results

Table 1 gives pressure values for each of the ten subjects analyzed, and summary statistics are given in Table 2. VC and LCR mean IAP values were 45.6 ± 4.65 and 44.5 ± 9.31 cm H_2_O, respectively (p = 0.05). Although the peak (maximum) IAP values for the LCR (164.9 ± 15.8) appeared greater than the VC peak IAP (139.5 ± 14.2 cm H_2_O), the difference was not significant (p = 0.07) (Table 2).

**Table 1:** AUC Values for Voluntary and Involuntary Reflex Cough.

**Subject**	**VC AUC (**cm H2O·s**)**	**RCT AUC (**cm H2O·s**)**	**VC mean IAP (**cm H_2_O**)**	**RCT mean IAP (**cm H_2_O**)**	**VC max IAP (**cm H_2_O**)**	**RCT max IAP (**cm H_2_O**)**
1	92	125	35.2	48.4	87	100
2	290	781	52.5	52.4	167	175
3	326	1063	31.6	49.8	100	170
4	375	1214	37.5	28.5	165	139
5	612	1308	70.2	58.9	211	174
6	483	1148	65.5	73.5	180	194
7	505	1239	55.0	50.8	139	173
8	488	1321	42.0	73.6	165	275
9	124	703	26.1	28.5	104	149
10	201	964	39.1	38.0	77	100

Subject 10 had a T_4 _complete spinal cord injury, and the IAP measurements were extremely low with a markedly compromised total cough force generated for VC and involuntary reflex cough as indicated by the AUC values of 22 and 111, respectively. As such, this subject was excluded from all statistical comparisons of VC and involuntary reflex cough.

**Table 2:** Statistical comparison between values for VC and for Reflex Cough.

**Variable**	**Unit**	**VC**	**LCR**	**P**
**AUC**	cm H2O·s	349.6 ± 55.2	986.6 ± 116.8	< 0.01
**Number of peaks**	-	1.78 ± 0.28	6.00 ± 0.94	< 0.01
Mean IAP *	cm H_2_O	45.6 ± 4.65	44.5 ± 9.31	0.052
**Peak IAP**	cm H_2_O	139.5 ± 14.2	164.9 ± 15.8	0.07
**Episode duration**	s	10.2 ± 1.36	27.0 ± 0.74	< 0.01

* Mean IAP calculated as a singular value for each cough event.

The number of peak pressures, duration of cough events, and AUC values were all significantly greater with the RCT relative to voluntary cough (Fig. 2 and Fig. 3; Table 2). The number of peak IAPs was greater for the LCR than for VC (6.00 ± 0.94 vs. 1.78 ± 0.28, p < 0.01), as was the episode duration (27.0 ± 0.74 s vs. 10.2 ± 1.36 s, p < 0.01). The mean (± SEM) AUC values for VC and the RCT were 349.6 ± 55.2 cm H_2_O·s and 986.6 ± 116.8 cm H_2_O·s (p < 0.01; Table 2), respectively.

**Figure 4:**
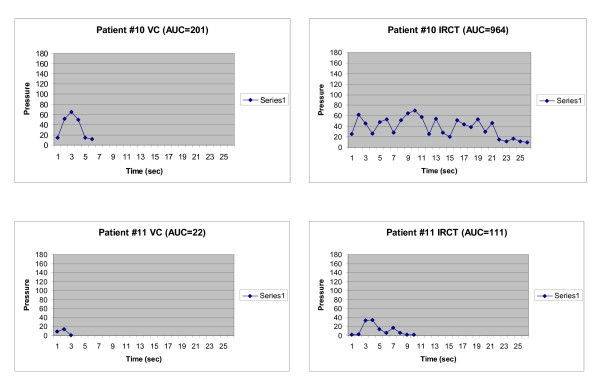
Area under the Curve Graphs for Subjects 10 and 11. Subject 10 had SUI and multiple sclerosis and subject 11 had a T_4 _complete spinal cord injury. This cohort group represents a sample of AUC values obtained from neurologically impaired subjects.

In the subjects with neurological impairment (Fig. 4), subject 10 had VC and RCT AUC values of 201 and 964 cm H_2_O·s, respectively (Table 1), and these normal values are included within the statistical analysis of Table 2. Subject 11, who had a T_4 _complete SCI, had VC and RCT AUC values of 22 and 111 cm H_2_O·s, respectively. When compared with responses in subjects without any history of neurological impairment, all of these parameters were decreased in the SCI subject (Fig. 4), but were similar to normal values in the non-ambulatory MS subject (subject 10).

The data from the SCI subject was not included in the statistical analysis due to their low magnitude. There were no adverse events experienced by the 11 subjects in this study.

## Discussion

The greater AUC value with the RCT, which triggers the laryngeal cough reflex [5,21], could be due to the continual and simultaneous activation of cough-associated expiratory muscles with rapid and repeated glottal closure, compared with VC with its brief and often single event of brief glottal closure (Addington et al. cited in [22]) [1,3]. The differences in the AUC between the two types of cough provide a new perspective to study the neurophysiological differences between these two events. Voluntary cough appears useful in clearing the vocal cords for speech and clearing the airways once material is present in the tracheobronchial tree; it seems similar to reflex cough from the tracheobronchial tree, which starts with an inspiration to increase lung volume. The LCR does not have an initial inspiration and is essentially a series of 'expiration reflexes' with intervening inspirations; it is for involuntary airway protection in response to a threatening stimulus [2]. The term "cough reflex" is often used generically to include both types of "cough" and also cough bouts or epochs.

The UD tracings indicated that the IAP appeared to be greater when there was no expiratory flow and the glottis was adducted. During VC and RCT cough, the IAP appeared to decrease when the glottis was abducted. However, during the coughing associated with the RCT episodes, the tracings showed a continuous increase in IAP above the initial baseline in all subjects, regardless of the duration of the cough episode and despite the subject having fully exhaled before initiating the LCR, which prevented any subsequent effective deep inhalation to assist the coughs. Although the LCR episodes may have had some brief inspiratory activity late in the epoch, the IAP remained elevated above the initial baseline throughout the entire event – this was a consistent finding irrespective of the number of expiratory efforts or the duration of the cough episode. Regarding neurological airway protection, we suggest that the main components of the LCR are primarily a continuous series of expiratory cough reflexes [6-8] with the possibility of some inspiratory efforts later in the epoch – what may resemble the initial stages of "true" cough. Thus, the continuously increased IAP during the duration of the LCR provides the sustained expiratory force for the protective airway function of the LCR. The neurophysiological status of airway protection appears to be appropriately assessed by the ability to measure the elevated intra-abdominal pressures over time.

The fact that peak IAP was usually greater for the LCR than for VC was surprising. The LCR was preceded by a forced exhalation before the RCT, and the VC was preceded by a forced deep inhalation before producing the VC. Videofluoroscopy clearly demonstrated the changes in the size of the thoracic cavity by the upward displacement of the diaphragm during VC and RCT (LCR) [1]. It is well established that the expiratory strength of cough is considerably greater when starting from a large lung volume compared with a small one [23,24]. The extent to whether this difference is due to the mechanical effect of stretched expiratory muscles, or to a lung reflex activated by lung inflation and enhancing the expiratory effort is debatable, although probably both mechanisms apply [1,22,25]. We did not measure lung volumes. The fact that the expected greater expiratory strength of the VC compared with the LCR was absent, even reversed, emphasizes a significant functional difference between the LCR and voluntary cough in this investigation. The AUCs were also much greater for LCR than for VC. Although this might be due to greater AUCs for individual expiratory efforts, this seems unlikely and the difference probably reflects the greater number and frequency of expiratory efforts for the LCR compared with the VC, with overlapping positive pressure curves. We cannot say if these differences also apply to cough from the lower airways.

Lasserson et al. demonstrated differences in muscle activation between voluntary and reflex cough [4]. Reflex cough from irritant chemical stimulation, assessed by surface electromyography (EMG), revealed simultaneous activation of all the expiratory muscles involved in cough, both primary and accessory. However, voluntary cough activated primary expiratory muscles first and then the contraction of accessory muscles occurred especially with a stronger voluntary cough effort. Their results for peak cough flow rates revealed that voluntary cough flow rate and the maximal cough flow rate achieved in any one effort was significantly higher for voluntary cough than for reflex cough. The involuntary cough results suggest that the glottis remains closed except for brief bursts of expulsive efforts. This would help to maintain the increased level of IAP found in our results and necessary for the next expiratory cough as well as to conserve lung volume until the threat to the airway has been resolved. The mean EMG duration of the voluntary cough effort was significantly longer than for reflex cough [4], but they did not consider the total expiratory electromyographic activity that occurs throughout the epoch of the LCR.

Lasserson's findings are important regarding the motor sequencing activation in voluntary compared with reflex cough [4]. Our experiments differ from theirs in that we may have used a stronger reflex cough stimulus, and more targeted to the larynx, with the aim of producing strong expiratory efforts. But it is clear from our findings that reflex cough can be assessed from one result of the stimulus, specifically an elevated mean intra-abdominal pressure over time. This pressure is sustained probably because the glottis is closed except for very brief episodes of abduction associated with the expiratory airflow. These series of expiratory coughs are essential for clearing the airway of a threatening supraglottic stimulus. The maintained closed glottis with the LCR explains our finding of elevated AUC values, and Lasserson's decreased peak expiratory flows with reflex compared with voluntary cough. The shorter EMG burst duration with reflex cough is modulated to maintain the elevated pressure without using too much pressure or losing the vital air needed to clear the airway over time. Since a forceful inspiration during airway clearing may result in aspiration of material into the lungs, if inspiration does occur during an episode of involuntary coughing, it is brief and appears weak. Voluntary cough peak airflows and EMG assessments are unreliable determinants of airway protection since their role is to clear rather than to protect the airways, and because the patient's participation can vary greatly. These considerations may have limited application to the usual cough from the lower airways, where large expiratory airflows may be necessary to remove material from the lungs.

We believe that you cannot determine involuntary neurological airway protection status from the assessment of voluntary cough. Involuntary cough function has multiple complex synchronous neurophysiological determinants that cannot be obtained from the assessment of voluntary cough. Voluntary cough is defined by deep inhalation followed by expiratory flows and expiratory pressures. Our data demonstrated that this is significantly different than expiratory (protective) cough reflex physiology.

Our definition of the LCR as it relates to neurophysiological airway protection in humans is the involuntary rapid synchronous expiratory muscle activation that causes and sustains an elevated intra-abdominal pressure event over time, sufficient for airway protection following a threatening supraglottic laryngeal stimulus [1-3,5,12,21]. The AUC values indicate that VC and the LCR are significantly different neurophysiological events. Quantification of the LCR using mean or peak IAP or AUC values may be useful as a clinical tool for determining the neurophysiological status of airway protection for an individual (Addington et al. cited in [22]).

## Authors' contributions

WA, RS, MP, RO were involved in the study design and protocol. RO was involved in the data collection. MP performed the statistical analysis and was assisted by JW. JW analyzed the data and significantly contributed to editing and writing the manuscript. WA and RS wrote much of the manuscript and were actively engaged in the interpretation of the results as was RO and JW.
